# Bioengineering Approaches to Accelerate Clinical Translation of Stem Cell Therapies Treating Osteochondral Diseases

**DOI:** 10.1155/2020/8874742

**Published:** 2020-12-24

**Authors:** Meng Wang, Yixuan Luo, Yin Yu, Fei Chen

**Affiliations:** CAS Key Laboratory of Quantitative Engineering Biology, Shenzhen Institute of Synthetic Biology, Shenzhen Institutes of Advanced Technology, Chinese Academy of Sciences, Shenzhen 518055, China

## Abstract

The osteochondral tissue is an interface between articular cartilage and bone. The diverse composition, mechanical properties, and cell phenotype in these two tissues pose a big challenge for the reconstruction of the defected interface. Due to the availability and inherent regenerative therapeutic properties, stem cells provide tremendous promise to repair osteochondral defect. This review is aimed at highlighting recent progress in utilizing bioengineering approaches to improve stem cell therapies for osteochondral diseases, which include microgel encapsulation, adhesive bioinks, and bioprinting to control the administration and distribution. We will also explore utilizing synthetic biology tools to control the differentiation fate and deliver therapeutic biomolecules to modulate the immune response. Finally, future directions and opportunities in the development of more potent and predictable stem cell therapies for osteochondral repair are discussed.

## 1. Introduction

Despite four decades of advances and achievements in the field of tissue engineering, reconstructing interfacial tissues such as bone-articular cartilage remains a significant challenge. Bone-articular cartilage, also known as osteochondral tissue, consists of cartilage, a calcified cartilage layer, and the subchondral bone with a proportion of 90%, 5%, and 5%, respectively. In severe traumatic incidents, both the cartilage and the subchondral bone are affected. Due to the ready availability and multipotent character, stem cells, especially mesenchymal stem cells, have become a focus in the field of osteochondral tissue engineering. However, the major obstacles to the construction of clinically useful osteochondral tissue are our inability to control the stem cell fate, differentiation to the extent needed, and the poor integration between engineered and host tissues after construct implantation.

In this review, we explore major clinical challenges for stem cell therapies toward joint preservation including administration and distribution, control of stem cell differentiation, and modulating a regenerative microenvironment. Under these challenges, we discuss several examples that leverage bioengineering approaches to improve stem cell therapies for osteochondral diseases. We will first discuss approaches of engineering biomimetic microenvironments to improve cell delivery and patterning, which include microgel encapsulation, bioadhesive inks, and 3D bioprinting to achieve accurate cell deposition and gradient living constructs. We will then discuss several examples of bioengineering strategies to engineer cells to control the differentiation fate and deliver therapeutic biomolecules to modulate the immune response. Finally, we will share our perspectives on the future endeavor to develop more potent and predictable MSC therapies.

## 2. Overcoming Clinical Challenges from Administration and Distribution

### 2.1. Challenges Associated with Local Administration and Distribution

The damage of osteochondral tissue is one of the major causes of osteoarthritis (OA), which is affecting the life quality of ~61.2 million people in China alone [1]. To repair and reconstruct osteochondral lesions for preventing OA progression, the replication of the innate physiological structure, function, and living milieu of cartilage and the subchondral bone is of significant importance. Currently, clinical strategies for the regeneration of osteochondral tissue such as abrasion arthroplasty, microfracture, and articular chondrocyte transplantation have received positive results in midterm follow-up periods. However, the long-term efficacy of these approaches is still unsatisfying due to the generation of fibrocartilage and poor integration between transplants and resident tissue [2]. In addition, adult cell-based therapies, such as autologous chondrocyte implantation (ACI), are restricted by cell availability and expansion potency. Stem cells, most commonly found in the embryo, bone marrow, adipose tissue, and synovium, have differentiation capability toward chondrocytes and osteoblasts under specific biochemical and biomechanical stimulation, which have offered a new platform for osteochondral regeneration and OA treatment in both preclinical and clinical situations. Local administration of MSCs, providing a straight path to the target site, is commonly utilized in OA clinical indications. For example, Mayo Clinic is using single and multiple injections of the culture-expanded autologous adipose-derived mesenchymal stromal cells (AMSCs) for investigating the safety and feasibility of treatment in mild to severe knee OA, which is currently in Phase I of the interventional clinical trial. Researchers from Ren Ji Hospital and the cooperating unit established a preclinical study to explore the efficacy and safety of human AMSC injection in intra-articular cartilage for the relief of OA symptoms (5∗10^7^ MSCs showed the best improvement) [3]. However, the therapeutic efficacy is still hampered; the dominant barriers are (1) the need for large dosages of cells (the dose normally ranges from 10^6^ to 10^9^ cells/injection), partly because of the short residence time after depositing to tissue sites [4, 5], and (2) the low cell survival rate caused by multireasons, including severe shear stress formed in the process of viscous hydrogel precursor injection [6, 7], hostile and immune microenvironment at the disease site, and insufficient nutrients and oxygen supply, which have been reviewed elsewhere [8]. In this section, we will first discuss different bioengineering strategies used to improve the survival rate and retention of stem cells in osteochondral lesions and further discuss the potential of employing various biomanufacture systems, such as 3D bioprinting, to accurately deposit stem cells and form gradient complex tissues.

### 2.2. Strategies to Improve Viability of MSCs by Encapsulation

Local administration of MSCs is a favored delivery approach compared to systemic delivery as it is easier to access the disease site and results in better therapeutic outcomes. However, insufficient retention and survival of transplanted MSCs at the diseased sites hampered its therapeutic efficacy. Using biomaterials to encapsulate MSCs is a promising approach to increase the retention and viability at the infarction site [9].

**Figure 1:**
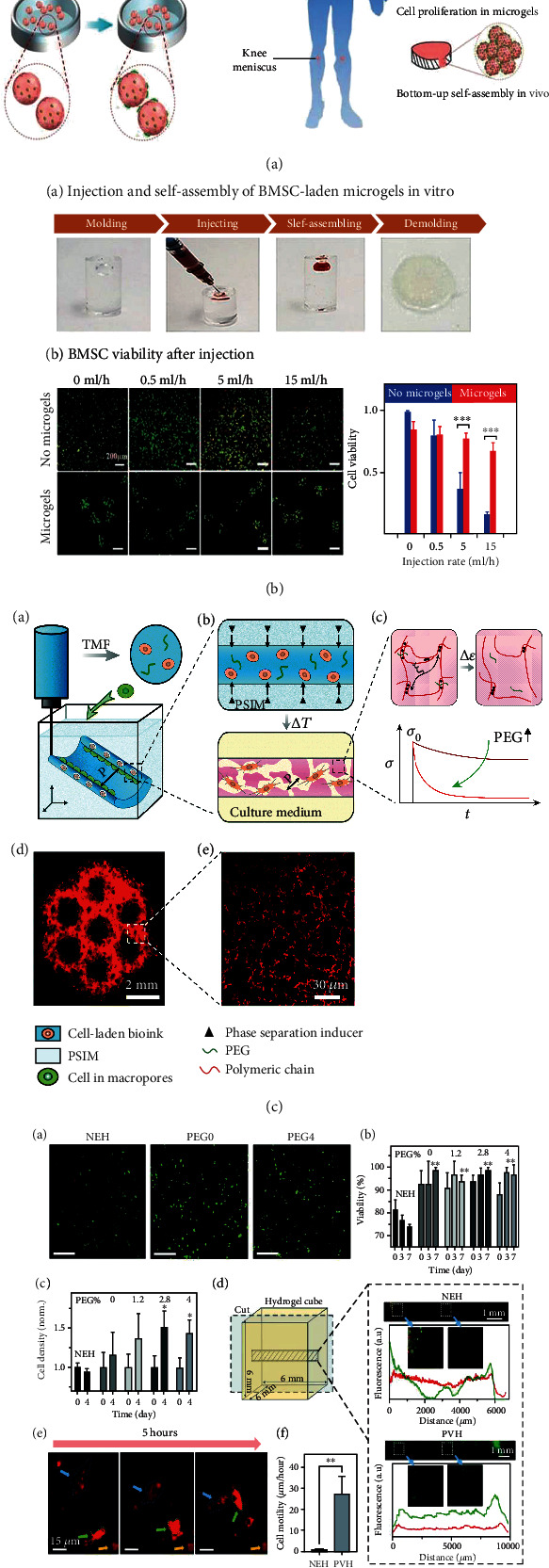
Encapsulation techniques for improving stem cell viability. (a) Microgel encapsulating stem cells can self-assemble into 3D microporous scaffold and (b) maintain high cell viability and chondrogenic differentiation potential. (c) Triggered micropore-forming bioprinting strategy improved cell viability compared to bulk hydrogel and (d) cell migration in micropore hydrogels. Reproduced with permission [13, 16].

Cell encapsulation within microgels (~1-1000 *μ*m) offers many advantages compared to encapsulation in bulk hydrogels [10] as it can supply an ECM-like 3D milieu for cell culture and expansion [11]; the micrometer-sized pockets of interstitial space between microgels can provide good diffusion of nutrients and oxygen [12]; most importantly, it can physically protect encapsulated cells from shear stress during injection. For example, under the same injection rate (15 ml/h), cell viability of BMSCs encapsulated in gelatin/hyaluronic acid hybrid microgels (67.5%) was higher than the medium suspended one (15%), meanwhile maintaining normal cellular functions, such as cell proliferation and chondrogenic abilities (Figures 1(a) and 1(b)) [13]. Similar phenomena were also observed in the bone regeneration study. In Hou and coworkers' work, poly(vinyl alcohol)-based microgels were developed for encapsulating MSCs and BMP-2 growth factor (GF) to induce osteogenic differentiation; high cell bioactivity and sustained release of GF were obtained by optimizing the crosslinking conditions of microgels, which ensured a specific and upregulated osteogenic differentiation and hence more efficient bone regeneration [14]. In terms of immune response, one interesting study found that geometry of implanted microgels could affect foreign body immune response and fibrosis in rodent and nonhuman primate models [15].

Although microgels can improve stem cell viability and enhance regeneration efficacy of cartilage and bone through physical protection and increased nutrition diffusion, the mechanical properties of assembled microgels are usually weak which cannot withstand physiological mechanical loads in osteochondral interfaces. Researchers recently reported a new strategy called triggered micropore-forming bioprinting (Figure 1(c)), to improve cell viability through microscopic pore formation in bulk hydrogel while preserving superior mechanical robustness (Figure 1(d)) [16]. The micropores were formed by temperature-triggered microphase separation and stabilized by hydrogen bonds of chitosan. Without sacrificing mechanical robustness, the bioprinted scaffold with interconnected pores (~17.8 *μ*m) supports cell spreading, migration, and proliferation. Impressively, the stiffness and viscoelasticity of the scaffold can be orthogonally controlled through a slight change of pH and the amount of PEG in the bioink. Though this system is yet to be applied in cartilage or osteochondral regeneration, it demonstrated potential of improving stem cell viability and maintaining mechanical robustness of porous hydrogel simultaneously.

Collectively, the approaches of microencapsulation and micropore-forming bioprinting may promote the efficacy of stem cell therapy via increasing the survival rate and reducing needed dosages of stem cells.

### 2.3. Strategies to Improve the Persistence of MSCs in the Host

Cartilage is surrounded by synovial fluid and acts as a load-bearing buffer which protects the bone and disperses shock and stress. To achieve the regenerative properties of stem cells, retention of injected stem cells at the dynamic defect site is vital for successful tissue regeneration. Strategies have been developed to improve stem cell retention in situ through different forms and types of biomaterials [17].

**Figure 2:**
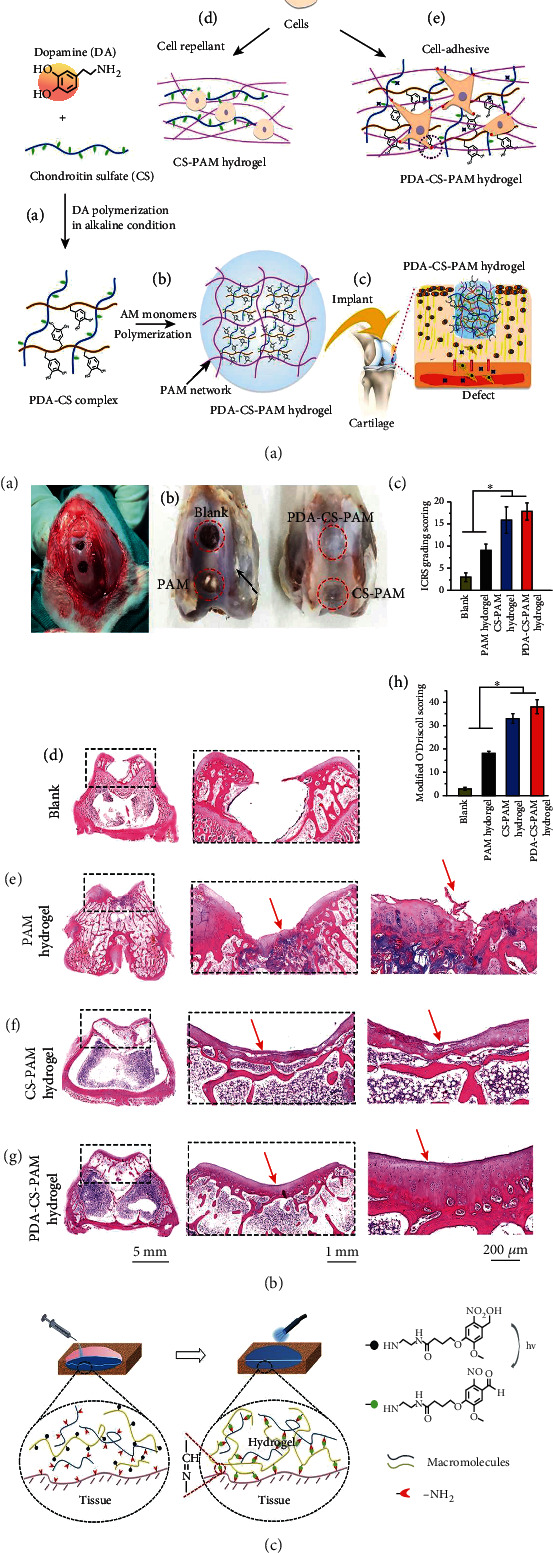
Adhesive hydrogels for improving the persistence of MSCs in the host. (a) Mussel-inspired tissue-adhesive cell carriers; (b) assessment of mussel-inspired tissue-adhesive cell carriers used in cartilage regeneration; (c) phototriggered tissue-integrable hydrogel developed for prolonging stem cell retention in situ. Reproduced with permission [18, 22].

One strategy is to incorporate mussel-inspired adhesives into the stem cell-based grafts. For example, Han et al. developed a polydopamine (PDA) modified chondroitin sulfate- (CS-) polyacrylamide (PAM) hydrogels with tissue adhesiveness for cartilage regeneration (see Figures 2(a) and 2(b)). The composite hydrogel exhibited good resilience and toughness because of the noncovalent interaction between PDA and CS and covalently crosslinked PAM network [18].

Similar to mussel-inspired catechol chemistry, gallol moieties which possess aromatic rings with three hydroxyl groups have recently been incorporated into cell-carrying hydrogels to achieve adhesive property. Shin et al. [19] developed gallol-modified ECM hydrogel inks which exhibited fast covalent crosslinking and tissue adhesion. The manufactured bioink maintained ~95% of cell viability after printing and can be printed on tissue substrates due to the adhesion of gallol groups to ECM. However, the mechanical property of gallol modified ECM hydrogel is much lower than the native cartilage. Furthermore, a high dose of the gallol group is cytotoxic to cells, which has been reported in previous research by the same research group [20]. Thus, the usage of gallol to synthesizing adhesive cell-loading inks should be further carefully evaluated in preclinical studies.

Another mechanism employed to prolong the residence time of stem cells is to develop a cell carrier with specific functional groups (e.g., aldehyde) which could react with amino groups on the surface of cartilage tissue. Zhou et al. studied an oxidized dextran- (ODex-) based construct for cartilage defect repair [21]. In this study, ODex not only make up the scaffold network via reacting with gelatin for superior mechanical performance but also formed good tissue adhesion by reacting with amino groups existing on the cartilage, which further promoted the integration of transplants and host osteochondral lesions. However, a high degree of aldehyde substitution to the dextran backbone is harmful to cells; thus, it is essential to shorten the duration of stem cells in the aldehyde environment. Yang et al. and colleagues prepared a novel phototriggered imine reaction to resolve the above limitations (see Figure 2(c)). In this system, o-nitrosobenzene (NB) can be transferred to NB-aldehyde under the exposure of 365 nm light and immediately crosslink with -NH_2_ in the polymer or surface of surrounding tissue [22]. This kind of phototriggered adhesive mechanism offers a good spatiotemporal control on cell viability, tissue adhesion, and tissue integration, providing a new strategy to prolong the retention time of stem cells in host tissue and facilitate seamless tissue integration for osteochondral regeneration.

In the future, there are opportunities to endow adhesive features to the microgel systems. It would be interesting and meaningful for endogenous repair as the adhesive microgels could adhere to the target tissue while facilitating endogenous cell infiltration to the microgel scaffold.

### 2.4. Accurate Cell Patterning to Improve Osteochondral Tissue Regeneration

Osteochondral tissue exhibits spatial gradients from the articulating surface to the underlying bone, with graded densities of chondrocytes, hypertrophic chondrocytes, and osteoblasts. These graded cell populations in osteochondral tissue secrete different ECM components that provide the tissue with spatial gradient mechanical properties to withstand the dynamic load-bearing environment. Thus, fabricating biomaterials and cell gradients to replicate the native gradient structures of osteochondral tissue is of critical importance in functional osteochondral tissue engineering [5].

3D bioprinting (3DBP), in which cells can be printed in either biomaterial-based or biomaterial-free bioink, has been extensively investigated in manufacturing desired topographies of osteochondral tissues [23, 24]. 3DBP uses multiheads to precisely deposit multibioinks; thus, diverse growth factors and cell types can be printed to the defect site according to the defined pattern and layer. For instance, a specific amount of MSCs and chondrocytes were accurately deposited in well-designed and printed microchambers to form cartilage, which possess comparable architecture, composition, and biomechanical performances to the native cartilage tissue according to histological, immunohistochemical, and mechanical analysis. Furthermore, this system was also applied in the building of entire artificial osteochondral tissue based on the construct of endochondral bone, which showed a stratified region of cartilage and bone by significant distinct content of GAG and calcium deposition [25].

**Figure 3:**
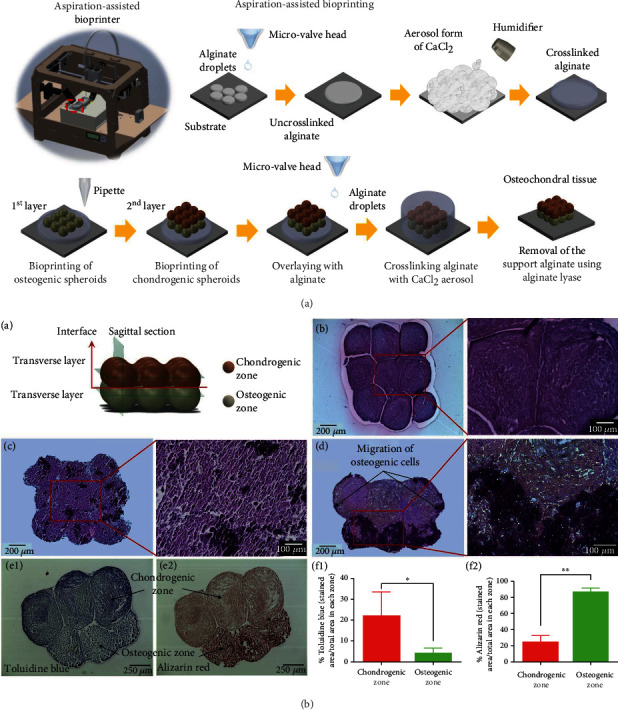
3D biomanufacture strategy used in stem cell accurate pattern to improve the efficiency of osteochondral tissue recapitulation. (a) Aspiration-assisted bioprinting process of osteochondral (OC) interface. (b) Characteristics of the bioprinted OC interface and the zone of cartilage and subchondral bone. Reproduced with permission [26, 27].

The inclusion of cells within the bioink is capable of directly fabricating defined cellular gradients, although spatiotemporal control of cell differentiation toward chondrocytes and osteoblast in bioink is still a big challenge. As an alternative, MSCs can be first differentiated into chondrogenic and osteogenic spheroids, respectively, and then accurately positioned to mimic the osteochondral structure. Ayan and coworkers recently developed a scaffold-free bioprinting approach to fabricate a dual-layered fused osteochondral interface through a homemade aspiration-assisted bioprinting (AAB) apparatus (Figure 3(a)) [26]. To reconstruct the osteochondral tissue, osteogenic spheroids and chondrogenic spheroids were first separately generated by the differentiation of human adipose-derived stem cells (ADSCs) in 3D culture. The OC interface was then bioprinted by first deposition of a layer of osteogenic spheroids onto a sacrificial support material (alginate crosslinked by CaCl_2_ vapor). Subsequently, another layer of chondrogenic spheroids was deposited onto a previous osteogenic layer. It is worth noting that the spheroids in individual layers can fuse together and the phenotypes in both zones can maintain through the study (Figure 3(b)) [27]. Similar to cell spheroids, different microgels encapsulating stem cells can be utilized as building blocks to form a predefined tissue with a spatial controlled cell type and gradient structure [12, 28, 29].

In short, biomaterial-based or biomaterial-free 3D bioprinting is promising in recreating gradient biochemical or biomechanical structures of osteochondral tissues. However, few challenges remain for applications of these artificial osteochondral constructs in the clinic. For example, Young's modulus [13] of most biomaterial-based stem cell implants is significantly lower than the natural articular cartilage (0.5-1.5 MPa) and bone tissue (15-20 MPa) [30, 31]. Integration force between engineered cartilage and subchondral bone as well as engineered osteochondral tissue with underlying native tissue is insufficient. In addition, facile and large-scale stem cell assembly techniques need to be developed for the creation of personalized constructs in clinic. Nevertheless, the precise assembly of stem cells with or without the aid of biomaterial provides a promising means to mimic the spatial complexity of the osteochondral tissue, which can be utilized not only in tissue engineering but also in drug testing and disease modeling.

## 3. Overcoming Clinical Challenges from Controlling Stem Cell Differentiation

### 3.1. Challenges Associated with Stem Cell Differentiation

The multidirectional differentiation potential makes MSCs become a sufficient source of seed cells for osteochondral tissue engineering and other disease therapies, but the precise control of cell differentiation in vitro and in vivo has been a huge challenge [32, 33]. The successful differentiation of stem cells involves many aspects, such as the interactions of MSCs to biomaterial scaffold, which we discussed in Section 2, biomolecular cues (growth factors, cytokines, trophic factors, etc.), and the applied culture systems [34, 35]. During developing an integrated multiphase tissue, crosstalk of signaling or potential interference between the different phases has a significant impact on the quality of engineered tissue. For instance, codelivery of genes of BMP-2 and TGF-*β*3 and to build an osteochondral construct may obtain insufficient cartilage forming or calcium deposition compared to individual delivery, because there can be antagonistic effects between chondrogenic and osteogenic growth factors [36]. In this regard, cellular engineering approaches including spatiotemporal control of transgene overexpression play increasing roles to control cell growth and differentiation, and we will examine few examples below to address these important questions.

### 3.2. Precise Control of MSC Differentiation with Spatial Gene Delivery for Osteochondral Regeneration

The repair of osteochondral defects involves the simultaneous regeneration of bone and cartilage. Correspondingly, MSCs need to be differentiated into chondrocytes and osteocytes. Traditionally, MSCs were, respectively, predifferentiated by the culture media containing specific growth factors. More recently, these bioactive proteins that drive the MSCs toward specific cell types have been directly embedded into the biomaterial scaffold. However, it is difficult to control the dose and spatial distribution of the growth factor in the scaffold device, and the proteins have a relatively short half-life in vivo [37, 38]. Therefore, gene delivery via viral or nonviral approaches has attracted considerable attention. Such genetic modification has the potential to produce high levels of expression of growth and transcription factors over long periods [39]. It is still difficult to, respectively, direct cell fate into certain lineages (i.e., cartilage and bone) using only one biomaterial scaffold from the same cell source, in a single culture system. To address this challenge, Huynh et al. [40] developed a method to induce osteogenic and chondrogenic differentiation of MSCs on two independent 3D woven PCL scaffolds in one single culture system. In general, TGF-*β*3 and BMP-2 are used to induce MSC chondrogenesis and osteogenesis, respectively [41, 42]. However, mothers against DPP homolog 3 (SMAD3) downstream of the TGF-*β*3 signaling pathway can repress Runt-related transcription factor (Runx2), which has great osteogenic capacity [43, 44]. Therefore, in this study, TGF-*β*3 was supplemented in a specific chondrogenic environment to promote the production of GAG and COL II on one scaffold. On the other scaffold, to inhibit the effects of chondrogenic-inducing TGF-*β*3 signaling, engineered MSCs overexpressing RUNX2 with knockdown of SMAD3 genes were prepared to generate a mineralized matrix in the same culture condition. This method could develop a bilayered scaffold with a layer of cartilage on top of a layer of bone below. In theory, the delivery of DNA plasmids coding tissue-specific inducing factors from multiphasic scaffold may be able to spatially facilitate cellular differentiation processes and further the regeneration of complex tissue structures [45]. Another research showed that the MSCs in different layers could also be induced to differentiate into chondrocytes and osteoblasts to form a bilayered osteochondral structure in vitro. This scaffold consists of one chitosan-gelatin scaffold layer activated by plasmid TGF-*β*1 for chondrogenic and the other hydroxyapatite/chitosan-gelatin scaffold layer activated by plasmid BMP-2 for osteogenic, respectively. And it was able to facilitate the regeneration of articular cartilage and subchondral bone in vivo simultaneously [46].

As a further attempt to spatially and temporally control the presentation of therapeutic genes to stem cells, Gonzalez-Fernandez and colleagues [47] developed a new pore-forming bioink combined with DNA plasmids encoding for either chondrogenic or osteogenic genes. By blending fast and slow degrading hydrogels, bioinks with increased porosity over time were achieved. The researchers found that the release rate of encapsulated pDNA in pore-forming bioink was higher than in solid inks; thus, it was possible to achieve transfection of transfected or host cells in either rapid and transient manner or slower and more sustained manner by modulating the porosity of these bioinks. Furthermore, these 3D bioprinted tissues could form a vascularized, bilayered, and stable osteochondral implant in vivo.

**Figure 4:**
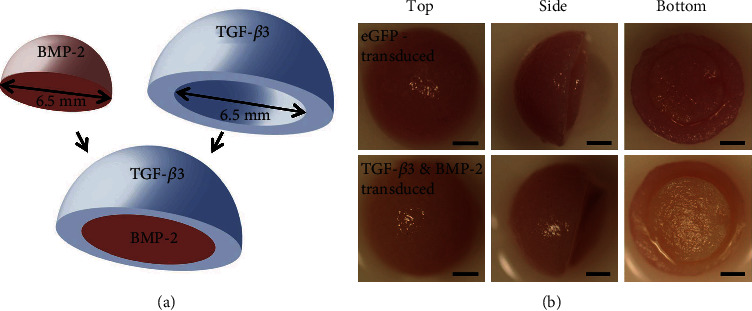
Osteochondral constructs. (a) Schematic diagram of fusing concentric hemispheres with embedded lentivirus of TGF-*β*3 and BMP-2. (b) Pictures of eGFP-transduced or TGF-*β*3+BMP-2-transduced constructs from 3 different angles. Scale: 2 mm. Reproduced with permission [48].

Another major challenge in developing successful constructs for osteochondral defect repair is to match the scaffold degradation rate with the neotissue formation. Toward this objective, Rowland and colleagues [48] recently developed constructs to suppress abnormal inflammatory response induced by the cytokine interleukin-1 (IL-1). They developed fusing concentric cartilage-derived matrix (CDM) hemispheres seeded with MSCs, respectively, overexpressing BMP-2 and TGF-*β*3 in addition with a doxycycline-inducible IL-1 receptor antagonist (IL-1Ra) transgene via the delivery of lentiviral particles. Their findings demonstrated that the gene delivery and the release of IL-1Ra effectively promoted the osteochondral tissue formation and protected the structure from degradation caused by the aberrant inflammation (Figure 4).

In summary, spatiotemporal delivery of therapeutic genes to locally control the differentiation of stem cells in vivo is a promising approach for the regeneration of osteochondral tissue. However, further effort needs to be focused on improving the transfection efficiency of the transfected cells and their immobilization at the site of action. Besides, the transgene combination and control of gene delivery need to be optimized to obtain better results during the tissue regeneration processes [49, 50].

## 4. Overcoming Clinical Challenges from Modulating a Regenerating Microenvironment

### 4.1. Challenges Associated with Host Factors

Although the administration and engineering of stem cells themselves are important to cell therapies, the host factors (local or systematic cytotoxic response, inflammation conditions, microenvironment, etc.) have also been shown to have a considerable influence on the biological fate and efficacy of stem cells in clinical trials [51]. For example, recipient cytotoxic response against the infused MSCs plays an important role in mediating the cell therapies. A study showed that hMSCs were phagocytized by monocytes after injected into a mouse model after 24 h, and this further promoted the immunotolerance by systemic immunoregulatory phenotype in the host [52]. The different stages and microenvironments of disease progression also can lead to the different effects of MSC therapies. During the progression of the disease, the internal microenvironment of inflammation, hypoxia, and many other pathological factors are dynamic, and it is difficult to take samples routinely from acutely ill patients [53]. Therefore, it is necessary to fully consider the impact of the host dynamic microenvironment on MSCs when applying them in therapies.

### 4.2. MSC Priming to Boost Their Potency toward Therapeutic Applications

Many studies have demonstrated that in order to exogenously boost the immunomodulatory function and clinical potency, MSC can be primed with proinflammatory cytokines or growth factors [54, 55]. For example, a soluble proinflammatory cytokine IFN-*γ* may affect adipogenesis and osteogenesis of MSCs [56]. Several IFN-*γ*-inducible genes such as Runx2 were found to upregulate during the early stage of osteogenic differentiation of BMSCs [57]. Besides, IFN-*γ* plays an important role in promoting the anti-inflammatory activity of MSCs. As reported, priming with IFN-*γ*, mouse MSCs (mMSCs) upregulated the expression of enzyme indolamine 2,3-dioxygenase (IDO), which has been shown to suppress T-cell activity in the early stage. And some important immunomodulatory molecules, including CCL2 PGE2, TGF-*β*, and HGF, were secreted from the primed mMSCs [58]. Another research suggested that the activation of the STAT1/STAT3 signaling pathway and inhibition of the mTOR signaling pathway facilitated the immunosuppressive properties of mMSC primed with IFN-*γ*. Also, the immunoregulatory ability was enhanced by the repression of the mTOR pathway in hMSCs [59]. As for the other cytokines, the alkaline phosphate activity and bone mineralization of MSCs were promoted when primed with LPS/TNF-*α* [60, 61]. Redondo-Castro et al. found that when treated with conditioned media of IL-1 primed MSC, murine BV2 cells secreted more trophic factors such as G-CSF and anti-inflammatory mediators such as IL-10, but less proinflammatory cytokines such as IL-6 and TNF-*α* [62]. Moreover, MSCs derived from AT, BM, or foreskin exhibited different expression levels of the immunoregulatory genes (IDO1, SEMA4D, FGL2, SEMA7A, and GAL) when primed with a proinflammatory cytokine mixture (IFN-*γ*, IL-1*β*, IFN-*α*, and TNF-*α*) [63, 64].

Since MSCs are highly sensitive to the harsh environment and will get function loss after cryopreservation during the preclinical or clinical trials, priming may help to improve the therapeutic potential of MSCs to target the biological properties of MSCs. In clinical translation, MSC priming still has many limitations, such as high costs, the harm of immunogenicity, unstable effects depending on the source and donor of MSCs, and the tumorigenic potential effect of MSCs treated with priming approaches during the long-term trials.

### 4.3. Engineered Stem Cells for Self-Regulated Drug Delivery Responding to an Inflammatory Environment

**Figure 5:**
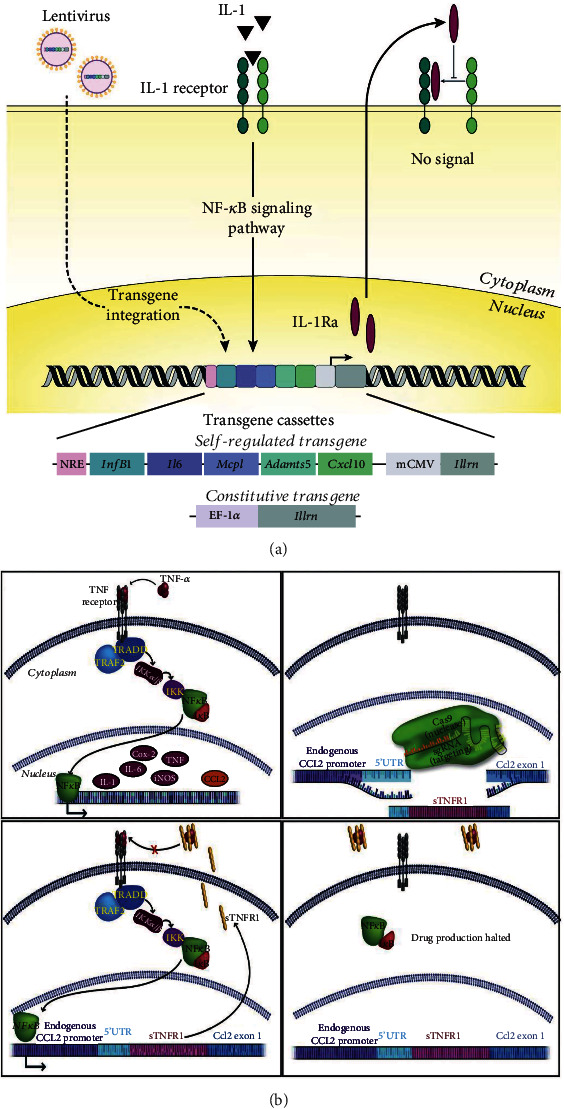
Synthetic gene circuits for self-regulated drug delivery systems. (a) IL-1 induced IL-1Ra expression through the NF-*κ*B signaling pathway. (b) sTNFR1 inserted in CCL2 loci can be initiated by TNF and inhibit TNF in return. Reproduced with permission [67, 70].

In addition to the osteochondral defect, both systemic and local inflammations may also have a profound impact on the pathogenesis of OA and other diseases. Although the proper pretreating with cytokines is beneficial to the immunomodulatory effect of MSCs, aberrant and continuously increased levels of proinflammatory cytokines such as interleukin-1 (IL-1), IL-6, IL-17, and tumor necrosis factor (TNF) can lead to the suppression of cartilage-specific genes and proteoglycan formation, in addition to the degeneration of the extracellular matrix (ECM). Furthermore, IL-1-mediated inflammatory environment inhibits chondrogenic differentiation of stem cells and leads to rapid degradation of cartilage derived from stem cells [65, 66]. Hence, there has been increasing investigations into therapeutics that may be beneficial in an inflammatory environment. Given the successful framework of a variety of protein therapies that are developed and applied in rheumatoid arthritis (RA), new approaches that edit the key transcripts of anticytokine molecules under endogenous promoter sequences have been applied to control the cellular response to inflammatory signals in the surrounding microenvironment dynamically. Pferdehirt et al. [67] developed a synthetic system using a designed promoter with several recognition elements of the nuclear factor kappa-light-chain-enhancer of activated B cells (NF-*κ*B) to amplify and induce the expression and release of anticytokine protein IL-1Ra. Transfecting the gene circuit into induced pluripotent stem cells (iPSCs) through lentiviral delivery, the engineered cells were capable of differentiating into engineered cartilage for the regeneration of diseased tissue and mitigating the inflammation in response to IL-1 in a self-regulated manner (Figure 5(a)). In recent studies, due to the highly targeted character and the low risks of tumorigenicity, the CRISPR-Cas9 system has revolutionized the applicability to mammalian cells [68]. A research showed that murine induced pluripotent stem cells (iPSCs) were engineered to functionally delete the IL-1 receptor I (*Il1r1*) using the CRISPR-Cas9 system. These modified cells produced more proteoglycan matrix and exhibited significant protection from the inflammation-induced tissue degradation compared to the wild-type cells [69]. Another similarly engineered iPSCs containing feedback-controlled gene circuits could be induced to produce bioactive drugs. Similarly, the base sequences expressing IL-1Ra or soluble TNFR1 (sTNFR1) were inserted downstream of the promoter of gene CCL2 to construct a dynamic negative feedback circuit activated by IL-1 or TNF using CRISPR gene editing (Figure 5(b)) [70]. During the latter research, the iPSCs in combination with a 3D PCL woven scaffolds were engineered to form a stable cartilaginous implant to alleviate the inflammation in a RA model [71]. The union of tissue engineering and synthetic biology promises a wide range of potential therapeutic applications for treating chronic diseases such as OA and RA by producing specially designed stem cells that not only can differentiate into tissue-specific cell types but can also regulate the expression of transgene molecules in direct response to dynamically changing pathologic signals in vivo. In the future, this highly responsive and self-regulated therapeutic strategy using designer stem cells for OA treatment could potentially overcome the limitations of traditional biologic anticytokine drugs or therapies and ultimately reduce the risk of adverse events in patients.

## 5. Conclusion and Future Perspective

Despite the fact that stem cells have provided tremendous promise to treat orthopedic diseases due to their inherent regenerative therapeutic and immunoregulatory properties, there still remain many challenges to realize their full therapeutic outcome, as increasing evidence indicated that in many cases, stem cells in their original state may not achieve the desired effect. Continued bioengineering approaches have improved the therapeutic efficacy; in particular, microgel assembly and 3D bioprinting techniques enable efficient cell encapsulation and improve cell survival and retention at the target site and precise cell patterning which mimics the gradient structure of the osteochondral interface. Coupled with other technologies such as transgene delivery, CRISPR-Cas9-based gene editing, designer stem cells that can dynamically modulate the extracellular environment can be functionally achieved. In addition, for successful applications of MSCs in clinic, certain biosafety concerns such as genetic abnormality, tumor formation, and induction of host immune response need to be carefully addressed. It is reported that genomic instability and mutation may be induced during continuous and long-term cell expansion. The potential tumorigenic risk of MSC treatments may be related to the aberrant cell phenotype and malignant transformation [72]. Besides, some MSCs may undergo malignant transformation in the recipients with immune deficiency or special tumor environment [73, 74]. Therefore, it is important to optimize culture duration and monitor the chromosomal karyotype and cell growth kinetics strictly during the manufacturing of MSCs using advanced cytogenetic techniques and miRNA analysis to avoid the risk of tumorigenicity. At last, MSC-related clinical trials should be based on substantial animal experimental studies confirming its safety and effectiveness.

In the future, we believe that bioengineering approaches will continue to profoundly influence the application of stem cell therapies for osteochondral and joint relevant diseases. Intelligent stem cell therapies with self-regulating capabilities for biologic drug delivery will be widely applied in OA and many other chronic diseases.
